# Understanding and meeting information needs for patients with posttraumatic stress disorder

**DOI:** 10.1186/s12888-016-0724-x

**Published:** 2016-02-01

**Authors:** Bradley V. Watts, Maha H. Zayed, Hilary Llewellyn-Thomas, Paula P. Schnurr

**Affiliations:** VA National Center for Patient Safety, White River Junction, VT USA; White River Junction VA Medical Center, White River Junction, VT USA; Geisel School of Medicine at Dartmouth College, Hanover, NH USA; The Dartmouth Institute for Health Policy and Clinical Practice, Lebanon, NH USA; National Center for PTSD, White River Junction, VT USA

**Keywords:** Shared decision making, Patient decision aid, Posttraumatic stress disorder, Patient-centered care

## Abstract

**Background:**

Posttraumatic Stress Disorder (PTSD) is a commonly occurring mental illness. There are multiple treatments for PTSD that have similar effectiveness, but these treatments differ substantially in other ways. It is desirable to have well-informed patients involved in treatment choices. A patient decision aid (PtDA) is one method to achieve this goal. This manuscript describes the rationale and development of a patient decision aid (PtDA) designed for patients with PTSD.

**Methods:**

We conducted an informational needs assessment of veterans (*n* = 19) to obtain their baseline information needs prior to the development of the PtDA. We also conducted a literature review of effective PTSD treatments, and we calculated respective effective sizes. A PtDA prototype was developed according to the guidelines from the International Patient Decision Aid Standards. These standards guided our development of both content and format for the PtDA. In accordance with the standards, we gathered feedback from patients (*n* = 20) and providers (*n* = 7) to further refine the PtDA. The information obtained from patients and the literature review was used to develop a decision aid for patients with PTSD.

**Results:**

Patients with PTSD reported a strong preference to receive information about treatment options. They expressed interest in also learning about PTSD symptoms. The patients preferred information presented in a booklet format. From our literature review several treatments emerged as effective for PTSD: Cognitive Therapy, Exposure Therapy, Eye Movement Desensitization Therapy, Selective Serotonin Reuptake Inhibitors, venlafaxine, and risperidone.

**Conclusion:**

It appears that the criteria set forth to develop decision aids can effectively be applied to PTSD. The resultant PTSD patient decision aid is a booklet that describes the causes, symptoms, and treatments for PTSD. Future work will examine the effects of use of the PTSD decision aid in clinical practice.

**Trial registration:**

Clinicaltrials.gov identifier NCT00908440. Registered May 20, 2009.

**Electronic supplementary material:**

The online version of this article (doi:10.1186/s12888-016-0724-x) contains supplementary material, which is available to authorized users.

## Background

### Posttraumatic stress disorder (PTSD)

Posttraumatic stress disorder (PTSD) is a common psychological disorder that can occur after exposure to a traumatic event such as military combat, sexual/physical assault, natural disasters, or vehicular crashes [1]. Symptoms of PTSD fall into four categories. These include intrusion symptoms (e.g., intrusive thoughts, nightmares and flashbacks), avoidance (i.e., avoiding thoughts, feelings, and people/places related to the traumatic event), negative alterations in mood (inability to remember, distorted cognitions, guilt), and arousal symptoms (e.g., irritability, trouble sleeping, hyper-arousal, and startle reflex) [1]. Individuals with PTSD often experience financial, interpersonal, occupational, legal, and/or housing problems [2]. PTSD can be a severe and disabling condition.

### PTSD prevalence

According to the National Comorbidity Survey–Replication, almost 7 % of adults in the United States (US) experience PTSD at some point in their lifetime [3]. The presence of PTSD is even more common in populations such as military combat veterans. According to the National Vietnam Veterans Readjustment study, 30 % of Vietnam veterans developed PTSD at some point following the war [4]. The Rand Corporation’s Center for Military Health Policy Research estimated that the current prevalence of PTSD is 14 % among veterans of the recent wars in Iraq and Afghanistan [5].

### PTSD treatment

PTSD treatment in clinical practice has been informed by several clinical practice guidelines that have been developed over the past decade [6–11]. Although the guidelines differ slightly in their recommendations, there is considerable consensus. Several psychotherapies appear effective (i.e., Cognitive Therapy, Exposure Therapy, and Eye Movement Desensitization and Reprocessing Therapy). Cognitive Therapies involve helping patients to restructure their maladaptive thoughts or thoughts that maintain their PTSD symptoms. The rationale is that such restructuring will help patients replace thoughts associated with fear with less distressing ones [12]. Exposure-based treatments involve having patients repeatedly re-experience memories of their traumatic event. The rationale is that this structured exposure to traumatic memories will help patients gain control of their emotional responses to their trauma and thus experience less fear [13]. Eye Movement Desensitization and Reprocessing involves having patients revisit their trauma memories while simultaneously performing saccadic eye movements. The rationale underlying Eye Movement Desensitization and Reprocessing Therapy is that coupling eye movements with revisiting of trauma memories will help patients gain control over their emotional responses to their trauma [14].

There is also consensus among most practice guidelines regarding medication [6–11]. The guidelines generally agree that the selective serotonin reuptake inhibitors (e.g., sertraline, fluoxetine, paroxetine) are an effective treatment. This class of medications affects the neurotransmitter serotonin and has strong evidence from randomized controlled trials supporting its use [15–18]. Similarly, selective norepinephrine reuptake inhibitors such as venlafaxine have been recommended by practice guidelines. This group of medications affects both serotonin and norepinephrine and has been shown to be effective in randomized controlled trials [19].

Although there is considerable evidence that a variety of treatments are effective for treating PTSD, there is no single best treatment. However, few head to head studies with adequate sample size have been conducted [20].

### Shared decision making

Current concepts of quality advocate not only for the use of effective treatments for a disorder, but also that the treatments be patient-centered [21]. Patient-centered care is a multidimensional construct that includes patient education and incorporating patient preferences into clinical care [22]. Shared decision making has been advocated as one method of achieving patient- centered care [23]. The term shared decision making refers to the communication that takes place with patients who wish to be involved in their health care decisions. Shared decision making is a method that addresses one of the Institute of Medicine core aims for improvement, patient-centered care [21]. Research suggests that shared decision making is associated with positive patient outcomes, such as increased knowledge, satisfaction with treatment, and improvement in symptoms [23].

### Patient decision aids

Shared decision making may include use of a patient decision aid (PtDA), which is a condition-specific tool designed to inform patients about treatment decisions. PtDAs can take the form of booklets, DVDs, videos, interactive computer programs, or websites [23]. PtDAs are specifically designed to help patients understand and choose between two or more relevant treatment options. Effective PtDAs do this by providing patients with detailed, high-quality, and balanced information about each option’s possible benefits and potential risks. The PtDA can help patients understand that the selection of a particular option is dependent on their informed preferences towards these benefits and risks.

The Cochrane Collaboration has systematically reviewed over 115 randomized clinical trials of PtDAs for use in screening, prevention, and medical-surgical care. The review confirms that well-designed PtDAs help patients to participate in making informed choices that are consistent with their values [23]. Moreover, PtDAs were better than treatment as usual when it came to increasing patients knowledge and satisfaction with treatment [23].

Recent research has examined the use of PtDAs for mental health disorders [24, 25]. In a study of patients with schizophrenia a decision aid resulted in enhanced patient knowledge of treatments [24]. Similarly a study conducted in patients with major depressive disorder found that patients’ use of PtDAs was associated with increased involvement in decision-making and satisfaction with mental health care.

In this paper, we describe the development of a PtDA for PTSD. At the time of this study, no PtDA for PTSD was available. PTSD is condition well-suited for a PtDA in that there are several effective treatments [20, 26, 27]. PTSD treatments do vary substantially in other ways. For example, a patient with PTSD could reasonably choose psychotherapy requiring a weekly visit with a therapist or medication therapy involving daily ingestion of a tablet at home. We conducted a randomized trial comparing the outcomes of patients who received the PtDa with patients who recieved usual care. We found that the decision aid led to greater knowledge about PTSD and less conflict about their choice of tretament, as well as increased likelihood of receiving evidence-based treatment and improved PTSD outcomes. In this paper, we describe the development of a PtDA for PTSD [28]. Below, we describe the three step process that we used to develop the PtDA: 1) informational needs assessment, 2) review of literature for descriptions of PTSD and corresponding effective treatments, and 3) prototype development.

## Methods

### General approach

This project was approved by the Dartmouth College Committee for the Protection of Human Subjects and registered on clinicaltrials.gov on May 20, 2009. Written informed consent was obtained from all participants.

We were guided by the International Patient Decision Aid Standards (IPDAS) collaboration criteria for both content and development of the decision aid for PTSD [23]. Briefly, IPDAS specifies that any PtDA should contain the following: natural course of condition without therapy, procedures for each treatment option, potential benefits and disadvantages for each option, and probabilities of benefits and side effects/harms. IPDAS criteria for development of a decision aid are as follows: based on current scientific evidence, conflicts disclosed, and field tests with patients and practitioners to ensure that the decision aid is acceptable and understood. The development of this new decision aid involved three steps that resulted in specifying the information content and developing the prototype.

### Step 1: Informational needs assessment

We recruited participants (*n* = 19) diagnosed with PTSD at the White River Junction Veterans Affairs Medical Center (VAMC). Participants could have a new or long standing diagnosis of PTSD. Participants were not excluded due to the presence or absence of any co-morbid medical or psychiatric diagnosis. A research assistant conducted a 60-min semi-structured interview with each participant. Participants were asked to report their opinions regarding (a) information they wished to know about PTSD, (b) information they felt they needed in order to make a therapeutic choice, and (c) their favored presentation format for information. We continued to recruit participants with PTSD to interview until consistent themes emerged among the participating veterans’ responses (i.e., theoretical saturation where no new themes emerge from participants and concepts in a theory are well-developed) [29].

### Step 2: Review of literature for description of PTSD and its effective treatments

No definitive review of PTSD treatments was available. Therefore, we conducted a comprehensive review and analysis of effective treatments for PTSD. We searched the relevant literature using PubMed, Medline, PILOTS, Psycinfo, and the Cochrane Collaboration’s databases from 1980 (the date of establishment of the Diagnostic and Statistical Manual criteria for PTSD) until January 1, 2013 [31]. The search terms “post-traumatic stress disorders,” “posttraumatic stress disorder,” “PTSD,” “combat disorders,” and “stress disorders, post-traumatic” were used [30]. The search results were limited to articles indexed as clinical trials or those that included the terms “treatment trial,” “randomized trial,” or “controlled trial” in their title or abstract. In order to locate additional sources, the authors systematically reviewed the references cited in all included studies as well as previous review articles or meta-analyses.

We considered only studies that (1) randomized participants to one or more active treatments and to a control group; (2) involved only adult participants who all met standard PTSD diagnostic criteria as set forth in the third, revised third, or fourth editions of the Diagnostic and Statistical Manual of Mental Disorders; and (3) included pre-treatment and post-treatment measures of PTSD symptoms [32–34]. Treatment trials of similar types of therapy were pooled into treatment groupings. Treatments were included in the decision aid only if: (1) they differed statistically from the control group; and (2) had been studied in 100 or more participants in all pooled trials (including the total number in the treatment and control groups).

We calculated an effect size, compared to control group levels, for each type of treatment [34]. For manuscripts that provided data regarding response rates, we also collected PTSD response rate. Treatment response was defined as no longer meeting PTSD diagnosis [35–37]. As most studies did not include the categorical data, we imputed missing values for treatment response based on the continuous outcome data. These response rates were then used to develop the graphical displays used in the PtDA.

For manuscripts that reported only a continuous measure of outcome, we transformed the between groups treatment effect size into a percentage response rate that approximated PTSD treatment response. This allowed us to derive a weighted, pooled, average response rate for each particular treatment grouping.

We reviewed several of the leading references in the field of PTSD in order to develop a description of PTSD and description of the different PTSD symptoms and treatments [7, 31]. The descriptions were modified as needed to reflect a desired seventh grade reading level.

### Step 3: Prototype development

We elected to pursue development of a decision aid in booklet format based on the feedback from the participants with PTSD, and we utilized an iterative design process for the drafts of the booklets. For each draft that was constructed, the booklet was reviewed by a committee that included experts in the field of patient decision aids and experts in the field of PTSD. The committee assessed whether the informational content met the International Patient Decision Aid Standards, met the informational needs of patients, and was consistent with the published literature.

After six iterative reviews cycles, an initial version was agreed upon. Patients with PTSD and providers who treated such patients were asked to provide feedback about the initial version. The focus of this field testing was to obtain reports about the clarity of the PtDA prototype. Participants (*n* = 20) with PTSD seeking treatment from the White River Junction VAMC were recruited and individually given the draft of the PtDA decision aid. They were asked for feedback regarding clarity of presentation, ability to hold attention, and overall balance. These participants were different from the initial group of veterans who participated in the needs assessment. All participants were diagnosed with PTSD and had been stable in treatment in the mental health clinic for at least two years. The participants were first allowed to review the draft version of the decision aid, and then reviewed each page with research assistant using a semi-structured questioning format.

In addition, psychiatrists and psychologists (*n* = 7) working in the PTSD treatment clinic were shown the decision aid and their feedback was sought. The providers were working in a specialized PTSD clinic. They were well-versed in all of the evidence-based treatments described in the PtDA. The provider group as a whole had extensive experience with the described PTSD treatments. Again, the feedback was collected through the same review process by a research assistant who conducted an interview guided by a semi-structured interview tool.

Feedback from both participant and providers was incorporated into a second version of the PtDA. An independent panel, who had not been involved in the previous development or review steps, reviewed the final version of the PtDA. Their feedback was collected and incorporated into the final decision aid version.

## Results and discussion

### Step 1: Information needs assessment

Nineteen participants (16 men, 3 women) completed the needs assessment interview. All participants were recruited from the mental health clinic at the White River Junction VAMC. Ages ranged from 28–66 years, with a mean of 48 years. There was considerable variability in the time they had been involved in treatment for PTSD ranging from one month to 34 years, with a mean of 5.6 years.

When veterans were asked: “What do you want to know about PTSD?” the majority (59 %, *n* = 11) wanted to know about PTSD symptoms or its long-term effects on health. For example, one veteran stated, “I don’t even know what the doctor means by PTSD…which of my problems are PTSD?” Another participant asked, “What I want to know is what this stress thing is doing to my heart.” Other issues that emerged with less frequency were: knowing that PTSD is treatable (21 %, *n* = 4); realizing that “it’s not my fault” (11 %, *n* = 2); knowing that PTSD is a mental illness (11 %, *n* = 2); and understanding that PTSD is a disorder that affects many people (11 %, *n* = 2).

Almost all participants (95 %, *n* = 18) also spontaneously reported that they wanted to know what treatments were available for PTSD and wanted a description of those treatments. One participant summed up many others thoughts in saying, “Skip all the crap, just tell me what works.” A majority (68 %, *n* = 13) also wanted to know about specific treatment efficacy. “They keep saying take such and such medication for PTSD, but how well does it work.” Other themes that emerged with less frequency included side effects (21 %, *n* = 4), availability of treatments (10.5 %, *n* = 2), and coverage of treatments by health insurance (10.5 %, *n* = 2).

When participants were asked: “How would you best like the information presented to you,” most wanted information presented in a booklet (47 %, *n* = 9) or presented verbally by a counselor or doctor (31 %, *n* = 6). “I want something that I can touch and hold,” as an example was reported by one participant. A smaller percentage of participants (21 %, *n* = 4) wanted information presented on the internet or via email. Other formats that emerged were video (10.5 %, *n* = 2), and verbal communication with veteran (5 %, *n* = 1). Please note three partisipants equally preferred two formats thus the total exceeds 19. There were no trends in the preferences based on either patient age or era of military service.

### Step 2: Review of literature for description of PTSD and its effective treatments

One hundred and twelve manuscripts examining 137 different treatment trials met the inclusion criteria for this review and were included in the analysis. Treatments included individual psychotherapy, group psychotherapy, pharmacotherapy, and other novel treatments for PTSD.

Several treatments showed a statistically significant improvement in PTSD symptoms compared to the control condition. The treatments that were both more effective than control and studied in more than 100 participants included psychotherapies (Cognitive Therapy, Exposure Therapy, and Eye Movement Desensitization and Reprocessing Therapy), and medications (selective serotonin reuptake inhibitors, risperidone, and venlafaxine). As these treatments showed clear evidence of effectiveness, they were selected for presentation in the draft PtDA.

A full description of the literature and meta-analysis can be found in Watts et. al 2012 [20].

### Step 3: Prototype development

The initial draft of the PtDA was twenty-one pages long. The first page was a cover page; the second page listed the goals of the PtDA, and then the PtDA itself consisted of five sections that provided a description of PTSD as well as information about its effective treatments. Specifically, information was provided about individual psychotherapy for PTSD, medications for PTSD, and finally a summary about the different types of PTSD treatment.

**Fig. 1:**
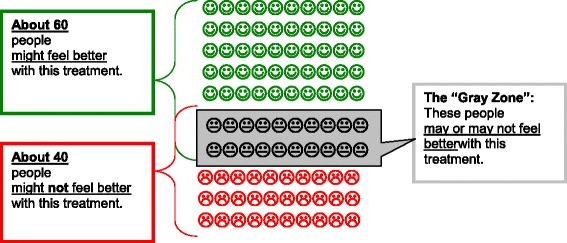
Visual depiction of treatment efficacy

We received feedback on the draft from 20 participants currently receiving treatment at the White River Junction VA. In addition, we received feedback from four psychiatrists and three psychologists. None of the participants or providers had been involved in the development of the draft. Participant and provider feedback was generally positive; respondents reported that the decision aid was “easy to follow,” and “easy to read and understand.” “The book seemed OK, it was pretty easy and helpful,” report one participant. “Another reported, “I think this would help a new veteran with PTSD pick a therapy.” The primary concerns voiced by both participants and providers were that the graphics used to illustrate relative efficacy were difficult to understand (i.e., Fig. 1). “I didn’t get the smiley faces,” was a common comment. In addition, multiple small changes were recommended. These typically involved clarification and simplification of the language.

## Conclusions

We developed a PtDA designed for participants with PTSD that reflected their informational needs and current knowledge about effective treatments (see Additional file 1 for complete version of the decision aid). This decision aid was developed using the established standards for the development of a patient decision aid [28]. Early feedback suggested that the PtDA was understandable to participants with PTSD and providers who treat PTSD. This suggests that an acceptable decision aid can be developed for participants with PTSD by using the standards in the field.

Based on the informational needs assessment, it was clear that participants most wanted to know about PTSD and the effective treatments for PTSD. This is consistent with previous research that suggests that patients want to be more involved in their mental health care (when compared to care for physical illnesses) [38]. The development of a PtDA for PTSD provides a first step in the goal of providing more patient-centered care.

Some limitations should be noted. First, the treatments described in this PtDA were limited to those that emerged as effective from review of the published literature. While these treatments are likely to be effective for PTSD, some participants may not have access to all the treatments described. Second, as this PtDA was developed using feedback from participants receiving care at a VA hospital, its applicability to other populations with PTSD is unclear. It is possible that veterans with PTSD have unique preference for information. Similarly, we received considerable feedback from participants who had already been treated for PTSD. It is possible that treatment naive patients have different needs and preferences. Third, we sampled a small number of participants and providers from a single PTSD clinic for feedback regarding the decision aid. It is possible this group of participants and providers may differ in important ways from the general population of patients with PTSD and providers who treat PTSD. In addition, we asked providers or participants to provide feedback about the PtDA rather than use it. It remains to be seen whether providers or patients will embrace such a tool. Likely substantial barriers would be faced to get providers to use the PTDA in typical practice. Future research should focus on better understanding these barriers.

The presence of the decision aid does not guarantee that care for patients with PTSD will be patient centered. We know little about how or if this tool will be used; however, its availability provides a possible route towards patient centered care which we think will be of great interest to clinicians and researchers.

Although the development of the PtDA is a necessary first step, many questions remain about the use and effectiveness of the PtDA for PTSD. Our randomized trial found that the decision aid led to great knowledge and better clinical outcomes [28]. Future research is needed to further evaluate the effect on patients’ knowledge and decision making. Similarly, we have only limited information regarding what effect the PtDA for PTSD will have on the processes of care (i.e. type of treatment received) and PTSD symptom outcomes. We have little understanding about how the PtDA could be incorporated into the care process for patients with PTSD. For example, when is the best time to give the PtDA to the patient, and whether it is important for the provider to review the PtDA with the patient? Similarly we currently have little knowledge about how patients would use the PtDA. Will patients review the entire 21 page booklet, or will they focus only on those pages describing treatments they are most interested in having? If these important questions can be successfully addressed, there is potential for the PTSD decision aid to facilitate patient centered care for patients with PTSD.

## Additional file

Additional file 1:
**Complete version of the decision aid.** (PDF 176 kb)

## Abbreviations

DVD: digital video disk
N: number
PtDA: patient decision aid
PTSD: Posttraumatic Stress Disorder
US: United States
VAMC: Veterans Affairs Medical Centers
