# Supplementary material for: Understanding and meeting information needs for patients with posttraumatic stress disorder
Source: BMC Psychiatry. 2016 Feb 1;16:21. doi: 10.1186/s12888-016-0724-x (PMC4736082; doi:10.1186/s12888-016-0724-x)
Supplement: Additional file 1: — Complete version of the decision aid. (PDF 176 kb) [file 12888_2016_724_MOESM1_ESM.pdf]

TREATMENT OPTIONS
FOR
POSTTRAUMATIC STRESS DISORDER

The purpose of this decision aid is to help you …

1. Understand what Posttraumatic Stress Disorder (PTSD) is.

2. Learn about the different treatment options for PTSD.

3. Compare these different treatments to each other.

4. Prepare you to talk with your provider about choosing a treatment that’s best for you

There are 5 sections to this decision aid.

Section 1

What is Posttraumatic Stress Disorder (PTSD)?

PTSD is a condition that can occur after you have been through a traumatic event.

A traumatic event is something frightening that you see or that happens to you.

During this type of event, you may think that your life or others' lives are in danger. You also may feel afraid or feel that you have no control over what is happening.

Anyone who has gone through a life-threatening event can develop PTSD.

These life-threatening events can include:

- Combat or military exposure
- Child sexual or physical abuse
- Terrorist attacks
- Sexual or physical assault
- Serious accidents, such as a car wreck
- Disasters, such as a fire, tornado, hurricane, flood, or earthquake

After the event, you may feel afraid and nervous. You may feel numb or cut off from other people. You may have upsetting memories of the event and nightmares about it. You may also avoid anything that reminds you of the event. If these feelings or experiences don't go away or they get worse, you may have PTSD.

What are the symptoms of PTSD?

Symptoms of PTSD can be upsetting. They may disrupt your life and make it hard to continue with your daily activities. For example, they can affect your relationships, work, and other aspects of living. It may be hard just to get through the day.

PTSD symptoms usually start soon after the traumatic event, but they may not happen until months or years later. They also may come and go over many years.

If the symptoms last longer than 4 weeks, cause you great distress, or interfere with your work or home life, you may have PTSD.

There are 4 types of symptoms:

1. reliving the event,
2. avoidance,
3. numbing, and
4. feeling keyed up…

1. *Reliving the event (also called "re-experiencing symptoms"):*

Bad memories of the traumatic event can come back at any time.

You may feel the same fear you did when the event took place.

- You may have nightmares.
- You even may feel like you're going through the event again. This is called a flashback.
- Sometimes there is a trigger: a sound or sight that causes you to relive the event.

2. *Avoiding situations that remind you of the event:*

You may try to avoid situations or people that trigger memories of the traumatic event.

You may even avoid talking or thinking about the event.

- A person who was in an earthquake may avoid watching television shows or movies in which there are earthquakes.
- A person who was robbed at gunpoint while ordering at a hamburger drive-in may avoid fast-food restaurants.

  A person who was guarding bridges in Iraq, may avoid driving on bridges.
- Some people may keep very busy or avoid seeking help. This keeps them from having to think or talk about the event.

3. *Feeling numb:*

You may find it hard to express your feelings.

This is another way that some people with PTSD avoid difficult memories.

- You may not have positive or loving feelings toward other people and may stay away from relationships.
- You may not be interested in activities you used to enjoy.
- You may forget about parts of the traumatic event or not be able to talk about them.

4. *Feeling keyed up (also called "hyperarousal"):*

You may be jittery, or always alert and on the lookout for danger.

This is known as hyperarousal. It can cause you to:

- Suddenly become angry or irritable
- Have a hard time sleeping
- Have trouble concentrating
- Fear for your safety and always feel on guard
- Be very startled by sudden noises when someone surprises you

Section 2

Treatments for PTSD

There are different kinds of treatments for PTSD. **There are two main types**:

- **Psychotherapy**: this involves talking to a therapist to get better. You may talk with a therapist alone; this is known as "individual therapy." Or you may talk with a therapist in a group with other people who have PTSD; this is known as "group therapy." Psychotherapy may be called "counseling" too.

- **Medications**: This involves taking a pill to get better.

Some people get psychotherapy and medication at the same time. Some people may take more than one type of medication. You may want to consult with your provider to determine which are the best options for you.

Which PTSD treatments are known to work best?

Several kinds of treatments for PTSD can help people feel better. In this decision aid, we will tell you about treatments that work the best for patients with PTSD. **These treatments include three types of individual psychotherapy and three types of medications.**

- The types of individual psychotherapy are cognitive therapy, exposure therapy, and Eye Movement Desensitization and reprocessing Therapy (EMDR).

- The types of medication are selective serotonin reuptake inhibitors (SSRIs; sertraline, prozac, and paxil), venlafaxine, and risperidone.

Other kinds of treatments may or may not work, but have not been studied enough to show how well they work for PTSD. These kinds of treatments include group therapy, other types of individual therapy, and other medications.

Combining Treatments

We will present the treatments individually, but sometimes people get both medications and psychotherapy. You may choose to have both medications and therapy. We are aware that this is commonly done. Let's understand what could occur:

- You could choose therapy alone.
- You could choose medications alone.
- You could choose to start medications and therapy at the same time.
- You try either medications or psychotherapy after trying the other treatment.
- You use medications to treat specific symptoms that don't respond to therapy or a first medication.

None of these methods are clearly the best thing to do. This is an area for you and your provider to decide what is best for you.

Which treatment is right for me?

There is no single "best" treatment for all people. Instead, people with PTSD are encouraged to learn about the different treatments and then choose a treatment they feel will most likely benefit them.

The purpose of this decision aid is to help you understand these different PTSD treatment options and then pick the treatment that you think will be best for you.

We will talk about these two main types of treatment in separate sections. Then you'll have a chance to compare the treatments to each other.

Section 3

What about *Psychotherapy* for PTSD?

First, we are going to talk about psychotherapy for PTSD in general. Remember, psychotherapy is sometimes called "counseling."

Then we will tell you about three psychotherapy approaches that have been carefully studied and shown to work:

- Cognitive Therapy,
- Exposure Therapy, and
- Eye Movement Desensitization and Reprocessing (EMDR) therapy.

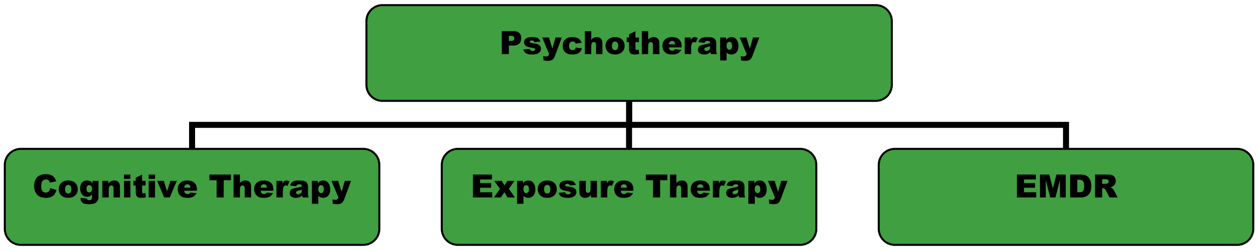

Psychotherapy in General

<u>Generally, psychotherapy for PTSD involves</u>:

1) Meeting for 60-90 minutes at a time, one-on-one with a therapist.
2) Meetings that are usually once or twice a week.
3) Meeting for at least ten sessions. Sometimes, there maybe more sessions, sometimes, there may be fewer sessions.
4) Realizing that sometimes, you will decide what you talk about; sometimes, the therapist may decide what you talk about.

<u>General factors to consider when deciding if psychotherapy is right for you</u>:

1) May require many visits
2) Frequent appointments
3) Is generally time-limited. You will usually meet for a fixed number of sessions.
4) Gains made during therapy usually remain even after you stop.
5) May talk about your traumatic experience
6) May have homework or practice assignments to do outside of the sessions

Now, we'll talk about each of the following in more detail:

- Cognitive Therapy
- Exposure Therapy
- Eye Movement Desensitization and Reprocessing (EMDR) therapy.

Cognitive Therapy

Cognitive therapy is a type of treatment in which you learn to change thoughts about the traumatic event that cause you stress. After a traumatic event, people may experience negative thoughts about themselves, others, and the world. For example, they may blame themselves for things they could not have changed. They may also experience feelings of shame and guilt. Cognitive therapy helps you understand that the traumatic event you lived through was not your fault by having you work through the way you think about and perceive the trauma.

With the support of your therapist, you engage in exercises that examine and challenge your thought process. One exercise involves having you write a narrative about your traumatic event. The goal is to understand how certain thoughts about your trauma cause you stress and make your symptoms worse. You will learn to identify thoughts about the world and yourself that are making you feel afraid or upset, and to replace these with more accurate and less distressing thoughts. You also learn to cope with feelings such as anger, guilt, and fear.

When deciding whether this treatment is right for you, there are several factors to consider:

1. This treatment requires meeting with your therapist once or twice a week.
2. This treatment requires you to talk about or discuss your traumatic experience.
3. This treatment requires you to complete homework or practice assignments outside of sessions.

Exposure Therapy

Exposure therapy is a type of treatment in which you talk about the traumatic event repeatedly, with a therapist, until you have less distress. You also do things in your daily life that you have been avoiding because they remind you of the trauma and make you upset. It is based on the idea that people learn to fear and then avoid thoughts, feelings, and situations that remind them of their trauma. While avoiding helps people feel better in the short term, it may also cause them to continue having symptoms of PTSD.

By talking about your trauma repeatedly with a therapist over time, you get an opportunity to work through your emotions related to the trauma. This will then result in your gaining more control of your thoughts and feelings about the trauma. For some people, it initially feels uncomfortable or difficult to think about the trauma or to do things they have been avoiding. But by not avoiding the memory and activities in your daily life you'll feel less overwhelmed over time. By working with your therapist, you gain skills to help you manage stressful memories and your symptoms.

Of **100** people, on average, with PTSD who go through Exposure Therapy, after 3 months, about **50 to 70** people report feeling better, and about **30 to 50** people report that they do not feel better.

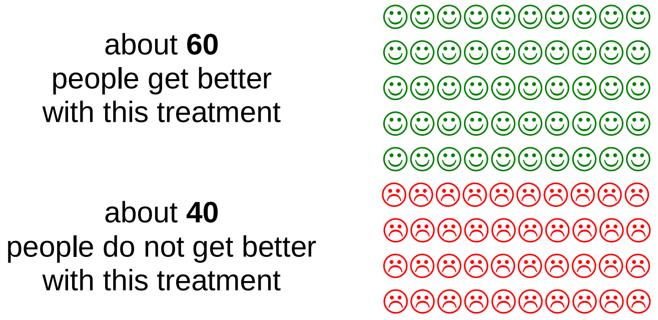

When deciding whether this treatment is right for you, there are several factors to consider:

1. This treatment requires meeting with your therapist once or twice a week.
2. This treatment requires you to talk about your traumatic experience in great detail.
3. This treatment requires you to complete homework or practice assignments outside of sessions.

Eye Movement Desensitization and Reprocessing (EMDR)

In EMDR, you focus on distractions like hand movements and sounds while you talk about the traumatic event. The idea is that the rapid hand movements make it easier for our brain to work through the traumatic memories. Over time, it can help change how you react to memories of your trauma.

The EMDR therapist begins by helping you learn skills that allow you to relax and handle emotional distress. These skills are used in the later sessions.

In the later EMDR sessions, you will select a traumatic memory or thought that bothers you. You then think about this memory or thought while you move your eyes back and forth in a way the therapist has taught you. For some patients who don't like the eye movements, the therapist can help you use other movements.

Of **100** people, on average, with PTSD who go through EMDR, after 1 or 2 months, about **25 to 45** people report feeling better, and about **55 to 75** people report that they do not feel better.

about **35**
people get better
with this treatment

about **65**
people do not get better
with this treatment

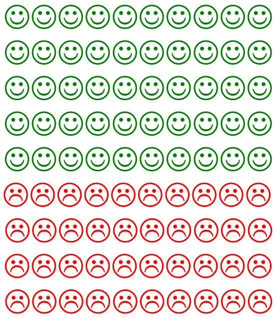

When deciding whether this treatment is right for you, there are several factors to consider:

1. This treatment requires meeting with your therapist once a week.
2. This treatment requires you to talk about your traumatic experience.

So what are the differences?

- In Cognitive Therapy, by talking with your therapist, you learn to change your thoughts about the traumatic event that cause you stress. Even though you talk about the traumatic event, there is less emphasis on the traumatic event compared to exposure therapy.

- In Exposure Therapy, you repeatedly talk about the traumatic event with a therapist, and engage in activities outside of session that you may have stopped doing since the traumatic event. There is more emphasis on talking about the traumatic event.

- In EMDR therapy, you focus on distractions like special movements and sounds while you talk about the traumatic event. There is less emphasis on the traumatic event compared to exposure therapy.

Now we’ll shift gears…

Section 4

What about *Medications* for PTSD?

We're going to talk about medications, which form the other group of treatments for PTSD.

[Remember three important points:

1. There are several PTSD treatments that work, but there is no single "best" treatment for all people.
2. The purpose of this decision aid is to help you understand these different PTSD treatment options, and then pick the treatment that you think will be best for you.
3. Even though we are presenting medications and therapy separately, sometimes, people choose to have both.

First, we are going to talk about medications for PTSD in general.

Then we will talk in detail about three types of medications that have been carefully studied and shown to work:

- Selective Serotonin Reuptake Inhibitors (SSRIs),
- Venlafaxine, and
- Risperidone.

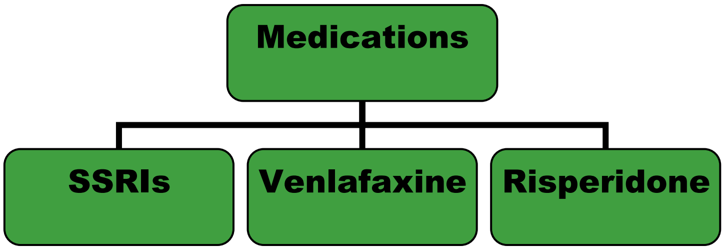

Medications in General

Generally, medication for PTSD involves:

1) You will take pills once a day
2) You will meet with a doctor about once a month
3) The medications do not work as soon as you start. However, they work as fast as psychotherapy/counseling

Before taking medications, there are two factors to consider:

1) Because the medication goes to all parts of your body, there is some possibility that you may have physical symptoms or side effects. Side effects can occur in fewer than 1 out of 3 people who take these medications. You need to consider the possible side effects when deciding if any medication is right for you:

2) Some of the medications used to treat PTSD may at times affect the way other medications you may take are working. You should consult your doctor about all the medications you are taking before you decide on a medication for PTSD.

Now, we'll talk about each of the following in more detail:

- Selective Serotonin Reuptake Inhibitors (SSRIs),
- Venlafaxine, and
- Risperidone.

Selective Seratonin Reuptake Inhibitors (SSRIs)

This a group of medications that includes Sertraline (Zoloft), Paroxetine (Paxil), and Fluoxetine (Prozac).

Each of these SSRIs involves taking a pill by mouth once a day.

Each of these SSRIs gradually affects the chemicals in the brain. These changes can cause the PTSD to improve or go away.

These SSRIs have similar possible side effects and success rates.

Of **100** people, on average, with PTSD who take an SSRI, after 3 months, about **35 to 45** people report feeling better, and about **55 to 65** people report that they do not feel better.

about **40**
people get better
with this treatment

about **60**
people do not get better
with this treatment

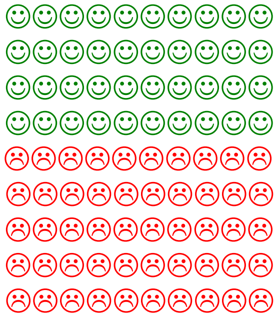

Side effects can occur in <u>fewer</u> than 1 out of 3 people who take this medication.
When deciding whether this treatment is right for you, you need to consider these possible side effects:

1. Nausea (feeling sick to your stomach)

2. Decreased interest in sex

3. Feeling drowsy, tired, or sleeping too much

Venlafaxine

Venlafaxine involves taking a pill by mouth once a day. Sometime patients will gradually increase the dose as they start the medication.

Venlafaxine gradually affects the chemicals in the brain. These changes can cause the PTSD to improve or go away. There are possible side effects from Venlafaxine.

Of **100** people on average with PTSD who take Venlafaxine, after 3 months, about **25 to 45** people report feeling better, and about **55 to 75** people report that they do not feel better.

about **35**
people get better
with this treatment

☺☺☺☺☺☺☺☺☺☺
☺☺☺☺☺☺☺☺☺☺
☺☺☺☺☺☺☺☺☺☺
☺☺☺☺☺

about **65**
people do not get better
with this treatment

☹☹☹☹☹☹☹☹☹☹
☹☹☹☹☹☹☹☹☹☹
☹☹☹☹☹☹☹☹☹☹
☹☹☹☹☹☹☹☹☹☹
☹☹☹☹☹☹☹☹☹☹
☹☹☹☹☹☹☹☹☹☹
☹☹☹☹☹

Side effects can occur in fewer than 1 out of 3 people who take this Medication.
When deciding whether this treatment is right for you, you need to consider these possible side effects:

1. Nausea (feeling sick to your stomach)

2. Dizziness

3. Feeling drowsy, tired, or sleeping too much

Risperidone

Risperidone involves taking a pill by mouth once a day. Sometimes patients will gradually increase the dose as they start the medication.

Risperidone gradually affects the chemicals in the brain. These changes can cause the PTSD to improve or go away. There are possible side effects from Risperidone.

Risperidone can be used alone or added to help another medication work. There is some evidence that it may work when other medications have not worked. You should discuss with your provider how Risperidone may work best for you.

Of **100** people, on average, with PTSD who take Risperidone, after 3 months, about **30 to 40** people report feeling better, and about **60 to 70** people report that they do not feel better.

about **35**
people get better
with this treatment

☺☺☺☺☺☺☺☺☺☺
☺☺☺☺☺☺☺☺☺☺
☺☺☺☺☺☺☺☺☺☺
☺☺☺☺☺

about **65**
people do not get better
with this treatment

☹☹☹☹☹☹☹☹☹☹
☹☹☹☹☹☹☹☹☹☹
☹☹☹☹☹☹☹☹☹☹
☹☹☹☹☹☹☹☹☹☹
☹☹☹☹☹☹☹☹☹☹
☹☹☹☹☹☹☹☹☹☹
☹☹☹☹☹

Side effects can occur in fewer than 1 out of 3 people who take this medication.
When deciding whether this treatment is right for you, you need to consider these possible side effects:

1. Muscle stiffness
2. Tremor or shaking of hands
3. Feeling drowsy, tired, or sleeping too much

We have discussed a number of treatments including therapy and medications which are best studied and been shown to help people with PTSD. Now we are going to discuss some medications that may be suggested to you.

These medications have not been as well studied and may or may not help PTSD. There may be some situations where these medications are discussed with you including:

- Other medications have not worked for you in the past
- You have certain PTSD symptoms that continue even after other treatments have been tried

For Example:

Prazosin may help with sleep.
Trazadone may help with sleep.
Quetiapine may help with sleep and intrusive thoughts.
Depakote may help reduce anger and irritability.

Again these medications are not well studied enough to recommend for everyone, but for certain people they may be helpful. Please discuss these with your provider if you have questions.

A few medications have not shown to help PTSD. These medications include

Benzodiazapines
Olanzapine

Section 5

Pulling It All Together

Now we'd like to summarize the main points about:

- the 3 types of psychotherapy/counseling and
- the 3 types of medications.

Three Types of Psychotherapy / Counseling

	Cognitive Therapy	Exposure Therapy	EMDR
**What is it?**	By talking with your therapist/counselor, you learn to change your thoughts about the traumatic event that cause you stress.	You repeatedly talk about the traumatic event with a therapist, and engage in activities outside of session that you may have stopped doing since the traumatic event.	You will focus on distractions such as special movements and sounds while you talk about the traumatic event.
**Number of sessions**	about 12	about 12	about 6 or less
**Efficacy**	Out of 100 people receiving this treatment about 60 get better & about 40 do not get better.	Out of 100 people receiving this treatment about 60 get better &about 40 do not get better.	Out of 100 people receiving this treatment get better, about 35 get better & about 65 do not get better.
**Factors to Consider**	• Weekly meetings<br>• *May require* you to talk about the traumatic event	• Weekly meetings<br>• *Requires* you to talk about the traumatic event	• Weekly meetings<br>• *Requires* you to talk about the traumatic event

Three Types of Medications

	**SSRI**	**Venlafaxine**	**Risperidone**
**What is it?**	Group of medications that affect brain chemicals	Medication that affects brain chemicals	Medication that affects brain chemicals
**Efficacy**	Out of 100 people receiving this treatment, about 40 get better & about 60 do not get better.	Out of 100 people receiving this treatment, about 35 get better & about 65 do not get better.	Out of 100 people receiving this treatment about 35 get better & about 65 do not get better.
**Factors to consider**	These occur in <u>fewer</u> than 1 out of 3 people who take this medication:<br>• Nausea (feeling sick to your stomach<br>• Decreased interest in sex<br>• Feeling drowsy, tired, or sleeping too much	These occur in <u>fewer</u> than 1 out of 3 people who take this medication:<br>• Nausea (feeling sick to your stomach)<br>• Dizziness<br>• Feeling drowsy, tired, or sleeping too much	These occur in <u>fewer</u> than 1 out of 3 people who take this medication:<br>• Muscle stiffness<br>• Tremor or shaking of hands<br>• Feeling drowsy, tired, or sleeping too much

Working with this decision aid is a basic first step to understanding PTSD and the available treatment options.

Your provider will be available to help you understand more, as you compare these different treatments to each other and consider their "pros" & "cons".

It's important to remember that the selection of a particular treatment is <u>your choice</u>.
